# Relationship between the Change in E/T Ratio and the Cooking Performance of Eucalyptus and Acacia Woods during Kraft Pulping Process

**DOI:** 10.3390/molecules28124637

**Published:** 2023-06-08

**Authors:** Jiangdong Yu, Xuewen Xu, Chen Miao, Penghui Li, Guolin Tong

**Affiliations:** Jiangsu Provincial Key Laboratory of Pulp and Papermaking Science and Technology, Nanjing Forestry University, Nanjing 210037, Chinaliph@njfu.edu.cn (P.L.)

**Keywords:** lignin, ozonation, spatial structure, kraft pulping

## Abstract

Lignin structure is an important factor affecting the cooking part of the pulping process. In this study, the effect of lignin side chain spatial configuration on cooking performance was analyzed, and the structural characteristics of eucalyptus and acacia during cooking were compared and studied by combining ozonation, GC-MS, NBO, and 2D NMR (^1^H-^13^C HSQC). In addition, the changes in the lignin content of four different raw materials during the cooking process were studied via ball milling and UV spectrum analysis. The results showed that the content of lignin in the raw material decreased continuously during the cooking process. Only in the late cooking stage, when the lignin removal reached its limit, did the lignin content tend to be stable due to the polycondensation reaction of lignin. At the same time, the E/T ratio and S/G ratio of the reaction residual lignin also followed a similar rule. At the beginning of cooking, the values of E/T and S/G decreased rapidly and then gradually rose when they reached a low point. The different initial E/T and S/G values of different raw materials lead to the disunity of cooking efficiency and the different transformation rules of different raw materials in the cooking process. Therefore, the pulping efficiency of different raw materials can be improved using different technological means.

## 1. Introduction

**Figure 1:**
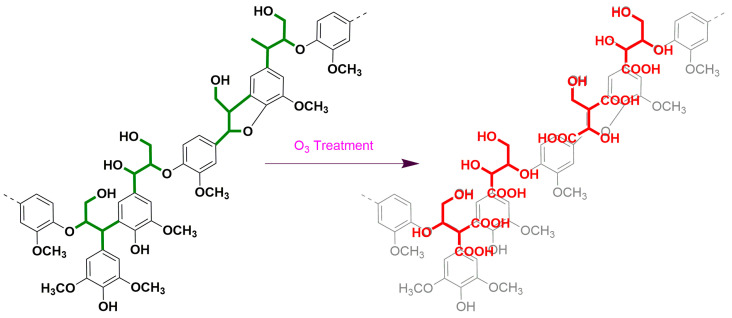
Principle of ozone reaction of lignin.

Lignin is one of the most abundant aromatic biopolymers and the main component of plant cell walls. It is mainly composed of the monolignols *p*-coumaryl, coniferyl, and sinapyl alcohols, which correspond to the *p*-hydroxypheny (H), guaiacyl (G), and syringyl (S) lignin units [1]. In addition, the arylglycerol-β-aryl ether linkage is the most prominent bond type connecting the phenylpropane (C_6_–C_3_) units of lignin [2], and this side chain structure is the source of the optical activity of lignin. The reaction principle of the oxidation of lignin macromolecules by ozone is shown in Figure 1. The selective degradation of the aromatic nuclei of lignin by ozone produced low-molecular-weight compounds, the resulting products retained the stereoscopic structure of the original stereoscopic side chain structure of lignin, and erythro(E) and threo(T) were formed. Meanwhile, the benzyl ether bond in lignin was rather stable during ozonation in acetic acid–water–methanol [3]. This method is not only suitable for soluble lignin in ozonation solvents, but also for insoluble lignin, such as lignin in wood powder or paper pulp, high polymer dehydrogenation polymer (DHP), Klason lignin, etc. However, this approach was initially limited by the fact that the quantities of erythro and threo formed during ozonation could not be recovered quantitatively, and the yields of erythro and threo were not always reproducible relative to the reproducibility of the internal standard.

With the advancement in technology, the ozonation method to determine the E/T ratio has improved rapidly and proposed two modifications [4]: one is to treat the ozonation product via sodium thiosulfate reduction, and the other is to treat the ammonium salt with silyl alkylation so as not to form lactones for gas chromatography (GC). Based on this, ozone oxidation is widely used to study the side chain stereospecific structure of lignin.

For decades, a lot of work has been devoted to understanding the stereostructure of lignin side chains and its effect on the related reaction properties of lignin. Erdtman et al. [5] first applied this method to the configuration analysis of phenyl-coumarin-type compounds, which are the main products of isobutenol dehydrogenation and condensation. Later, Akiyama et al. [4] applied this method to analyze various side chain structures of lignin and proved that the core structure of lignin, namely arylglycerol-β-aryl, has racemic properties and optical rotation [6].

The β-*O*-4 structure of lignin is usually composed of two diastereomers: erythro(E) and threo(T) forms. In addition, by studying the β-*O*-4 structure of the lignin of birch wood meal, the β-ether structures are essentially racemic [7]. When the lignin content was lower, the syringyl/guaiacyl(S/G) ratio of lignin was higher and so was the erythro/threo(E/T) ratio [8]. Alternatively, Shimizu et al. [9] used this method to study the reactivity of lignin during alkaline delignification, which was quantitatively investigated focusing on the effect of the structural differences between syringyl and guaiacyl aromatic nuclei and between erythro and threo in the side chain of a β-*O*-4-type lignin substructure on the β-*O*-4 bond cleavage rate. The erythro isomers degraded faster than the corresponding threo isomers in all of the compounds. The S/G ratio is closely related to the E/T ratio [10] and the relationship between the stereochemical properties of the β-*O*-4 structure and the process of lignin formation is discussed [11]. The applicability of this insoluble sample has great advantages over other spectroscopic methods of soluble lignin using separation methods, as separation is usually not quantifiable and is always accompanied by changes in chemical structure.

The main mode of bond breaking during lignin degradation is ether bond breaking [12]. When delignified by alkali cooking, not only the total yields of erythronic and threonic acids of the residual lignin decrease, but the E/T and S/G ratios also decrease with the progress of delignification [13]. In alkaline hydrolysis, the erythro *γ*-hydroxyl of lignin may participate in the reaction and result in a relatively high E/T rate ratio [14]. The erythro structure of lignin hydrolyzed about 30% faster than the threo isomer [15]. However, during the reaction between reactive oxygen species and lignin, the α-hydroxyl and phenolic hydroxyl groups of the E-type structure are more acidic than those of the T-type structure, and the E-type structure is more likely to be negatively charged at these two positions [16]. This leads to a greater electrostatic repulsion of reactive oxygen species when reacting with them, resulting in a higher reaction rate of T-type units than E-type structures. During the reaction between lignin and basic hydrogen peroxide [17], the free radicals generated are not significantly selective for the stereoconfiguration of the lignin reaction. During the oxidation reaction [18], no significant reaction bias regarding the specific stereospecific structure was observed for the diastereoisomers of the lignin model compounds with non-phenolic β-*O*-4 structures (erythro- and threo-type structures) after treatment via hypochlorite or chlorite systems in alkaline, neutral, or acidic environments. Unlike the reactions in the cooking process, lignin is not stereospatially selective under some reaction conditions, and even reactivity opposite to it can occur under some specific conditions. This reveals another aspect of the reason for the difference in the reaction properties of the two structures of lignin. In modern pulping process production, the kraft pulping method is currently the most widely used. Lignin plays a key role in determining the quality of pulp production. Lignin removal from lignocellulosic biomass remains challenging and the use of chemical means to remove lignin affects the final pulp yield [19]. Lignin removal is an expensive process and the pulp and paper industry often offsets the cost by using lignin waste as biogas and biofuel [20]. Therefore, understanding the behavior of lignin during kraft pulping is essential for cooking process optimization and pulp quality improvement.

## 2. Results and Discussion

### 2.1. Chemical Composition Analysis of Raw Materials

#### 2.1.1. Analysis of Components

The chemical compositions of the four different raw materials are shown in Table 1. Total lignin contains acid-insoluble lignin and acid-soluble lignin. Among the four raw materials, the lignin content of the two acacia woods (IA and VA) is basically the same, and the total lignin content is around 24%, with slightly higher total lignin in AE and slightly lower total lignin content in CE. Among the raw materials, carbohydrates are mainly cellulose and hemicellulose [21], and both acacia and eucalyptus are hardwood; so, the polysaccharides in them are mainly glucan and xylan [22]. The glucan content of the four raw materials is basically not very different, being around 43%. In particular, the glucan content of IA was higher than the other three raw materials, at 49.37%. The content of total sugars was around 60%, where the content of CE was slightly lower and the content of IA was slightly higher. Additionally, the test results showed that the acacia wood was basically free of arabinan, and the content of arabinan in eucalyptus wood was also extremely low, which is consistent with the basic characteristics of hardwood [23].

#### 2.1.2. FTIR Spectroscopy

**Figure 2:**
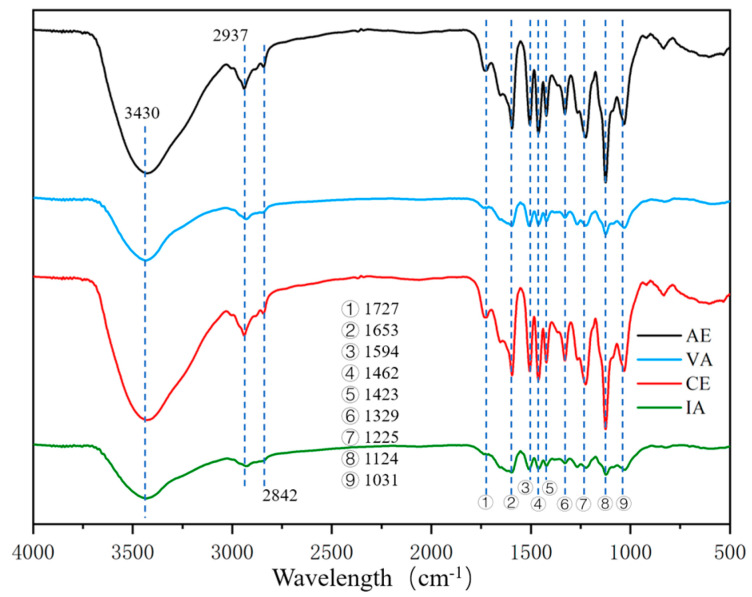
FT−IR spectra of MWLs from four raw materials.

The chemical structure of MWLs extracted from four kinds of raw materials was analyzed via FT-IR. It is well known that the structure of lignin in hardwood is mainly composed of high-content G units, low-content S units, and extremely low-content *p*-H units [24]. Hardwood and gramineous plants showed different G/S/H ratios. For hardwood species, different tree species also produced differences. These structural differences could be seen in the lignin of the four materials studied. The FTIR spectra of the four raw materials are shown in Figure 2. The allocation of major absorption bonds is shown in Table 2.

The absorption intensity at 1267 cm^−1^ was weaker for both AE and CE lignin samples, which indicates that the G-unit structure accounts for less of the structure of AE and CE. In contrast, for VA and IA, the absorption peaks of both S and G units are basically the same. In particular, since IA and VA were both acacia wood, the intensity of each absorption peak of the spectrum was basically the same, and the situation was similar for CE and AE. There was no difference between acacia and eucalyptus, in that the ratio of absorption peak intensity between the S-unit and G-unit of acacia wood was lower than that of eucalyptus. This indicates that there were more S-units in the basic units of eucalyptus wood, which is consistent with the results of nitrobenzene oxidation and 2D HSQC NMR analysis.

#### 2.1.3. ^1^H-^13^C HSQC NMR Analysis

**Figure 3:**
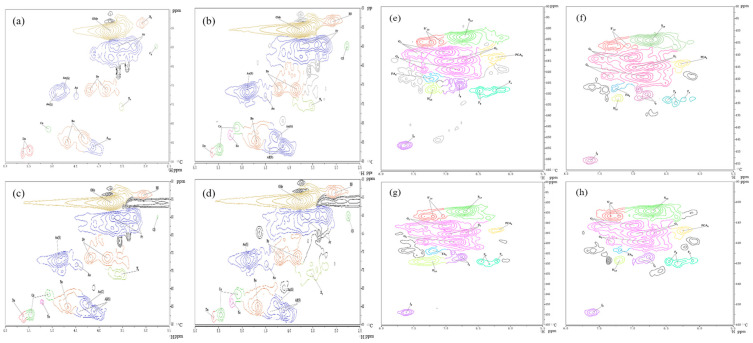
^1^H-^13^C HSQC NMR spectra of MWLs from four raw materials. The side chain area: (**a**): AE; (**b**): CE; (**c**): IA; (**d**): VA. The aromatic area: (**e**): AE; (**f**): CE; (**g**): IA; (**h**): VA.

**Figure 4:**
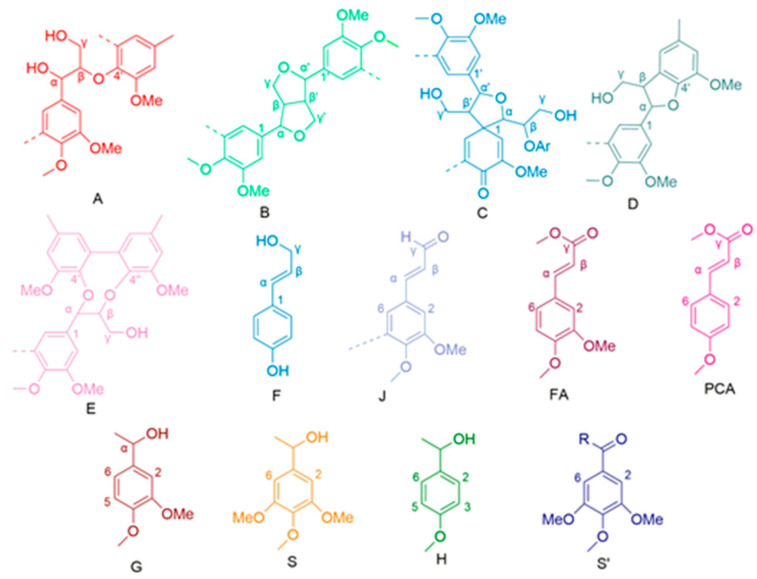
Main linkages and structures of lignin. (A) β-*O*-4′ linkages; (B) resinol substructures formed by β-β′, *α*-*O*-*γ*′, and *γ*-*O*-*α*′ linkages; (C) Spirodienone substructure; (D) Phenylcoumaran substructures formed by β-5′ and α-*O*-4′ linkages; (E) Dibenzodioxocin substructure; (F) *p*-hydroxycinnamyl alcohol end-group; (J) Cinnamyl aldehyde end-group; (FA) Ferulate; (*p*CA) *p*-coumarate; (G) Guaiacyl unit; (S) Syringyl unit; (H) *p*-hydroxyphenyl unit; (S′) oxidized syringyl unit.

^1^H-^13^C NMR is a powerful tool for probing the structure of lignin and its derivatives [25]. These signals are related to structural units and the various associations between lignin units in 2D NMR spectra can be assigned according to the published literature [26,27,28,29,30,31]. The NMR spectra of the four raw materials (δ_C_/δ_H_ 39.5/2.50) are shown in Figure 3. Table 3 describes the main lignin substructures shown in Figure 4. Semi-quantitative analysis based on the 2D HSQC signal was performed using Bruker’s Topspin 2.1 processing software, and the integration method was adopted using the method described in previously published papers [32,33].

As shown in Figure 3, the side chain region of the NMR spectra provided important information about the bonding patterns between different units in the structure of the lignin polymer. The NMR spectra of the lignin samples from different raw materials all showed strong signals from the lignin polymer features, as well as some weaker aliphatic and sugar signals, which indicated the high purity of the experimentally produced lignin samples.

As seen in Figure 3, the signals of the β-O-4′ aryl-ether bond (A) are very obvious. The δ_C_/δ_H_ correlation signals of C_α_-H_α_ in the β-O-4′ substructure are 71.94/4.88 and 71.04/4.79, respectively, which are mainly linked to G- or S-type lignin units. The δ_C_/δ_H_ correlation signal of C_β_-H_β_ in the β-O-4′ structure (linked to the G-type lignin unit) was 84.10/4.29, and the δ_C_/δ_H_ correlation signal of C_β_-H_β_ in the β-O-4′ structure (linked to the S-type lignin unit) was 85.90/4.13. The δ_C_/δ_H_ correlation signal of C_γ-_H_γ_ in the β-O-4′ structure was 59.78/3.63 and 3.25, and there is an overlap of signals with the others. In the aromatic area, signals from guaiacyl (G) and syringyl (S) are clearly observed in the MWL isolated from the four raw materials, indicating that the lignin type of all four raw materials is GS lignin, an essential characteristic of hardwood timber species. For the hardwood species, the signal from *p*-hydroxyphenyl (H) is clearly not as strong as gramineae.

Table 4 shows that the major substructures of MWLs are β-*O*-4′ alkyl-aryl ethers (IA, CE, VA, and AE are 69.72%, 65.61%, 61,96%, and 60.20%, respectively), while other linkage bonding types exist in small amounts as condensed structures (β-1′ spirodienones, β-5′ phenylcoumaran, β-β′ resinol). In particular, the contents of β-1′ spirodienones and β-5′ phenylcoumaran bonding types are essentially similar, while the contents of β-β′ resinol are significantly higher than those of the other condensed structures, which is consistent with the basic characteristics of hardwood species. For IA and VA, the lignin monomer structure content of the S-unit-type is relatively low, and thus the S/G ratio is also significantly lower than that of CE and AE. Since the E/T value shows a linear correlation with the S/G ratio, it can be concluded that the E/T ratio of eucalyptus is also higher than that of acacia. This is consistent with the test results of the ozone experiment performed earlier.

The S/G ratios of AE, CE, IA, and VA were 3.40, 2.03, 1.20, and 1.28, respectively, and the percentage of S-unit content in the lignin of eucalyptus raw material was significantly higher than that of acacia. The resinol structures of AE, CE, IA, and VA accounted for 26.19%, 13.86%, 22.18%, and 27.17%, respectively, and this structure generally represents the β-β′ bonding type of linkage between lignin units. The resinol is the most important non-ether bond for the linkage patterns between different lignin monomers. During the growth of eucalyptus, the S-type lignin units generally increase with the age of the tree and this structure does the same; in other words, the structural share of resinol and the share of S-type lignin units are positively correlated.

### 2.2. Analysis of Cooking Efficiency

**Figure 5:**
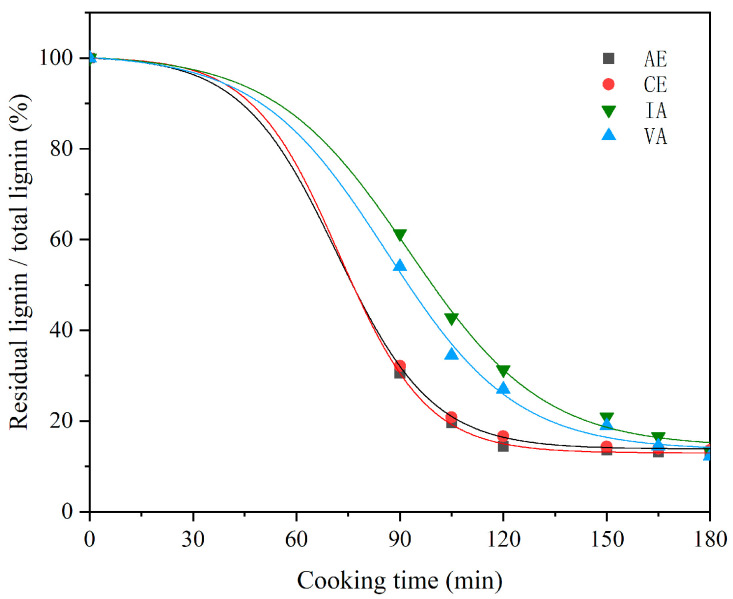
Curve of the variation pattern of residual lignin content with cooking time.

In the cooking process, lignin removal is mainly divided into three stages: initial delignification stage, massive delignification stage, and residual lignin removal stage. As the cooking time goes on, the content changes in the four raw materials of lignin are shown in Figure 5. They have the same rule as the conventional kraft cooking pulping, and go through three stages of lignin removal. The initial delignification stage mainly occurs in the period from the beginning of the temperature rise to 140 °C. In this stage, the liquid chemical begins to gradually saturate into the raw material, and the dissolution and removal of lignin are basically little. A large amount of the delignification stage generally refers to the initial stage, from the initial cooking temperature at 140 °C to heat preservation, and during which there will be a large amount of lignin dissolution. The removal stage of residual lignin refers to the removal of the little residual lignin left in the late cooking stage, and less lignin is removed in this stage. Based on this basic rule, the four raw materials are divided into two groups, eucalyptus and acacia, showing some significant differences.

For two types of acacia (IA and VA), the delignification rate gradually increases after heating, gradually approaches the maximum value after 140 °C, and reaches the maximum value after 30 min of heat preservation. With the gradual decrease in lignin content in raw materials and the decrease in alkaline liquid concentration in the late cooking liquid, the lignin removal rate decreases slowly. Finally, the delignification rate is almost 0 at 75 and 90 min of heat preservation. In a word, for IA and VA, a large amount of lignin removal from the raw materials occurs in the stage from 140 °C to 170 °C and in the stage from 170 °C to 170 °C for 60 min after heating.

For two types of eucalyptus, CE and AE, the overall efficiency of delignification shifted forward. During the period from 80 °C to 140 °C, the delignification efficiency of these two kinds of wood was not different from that of the previous two kinds of acacia wood. This is because, in the cooking process stage, the temperature is a very critical condition. When the cooking temperature does not reach the reaction condition, the change in the cooking device is mainly the penetration of the liquid chemical into the raw material. When the temperature reaches 140 °C, some changes happen. Unlike the two kinds of acacia wood described previously, the lignin removal rate of eucalyptus wood from 140 °C to 170 °C gradually approaches the maximum value, and finally reaches the maximum value at 15 min. Especially for AE, this time point may be a few minutes further ahead. In conclusion, for CE and AE, a large amount of lignin removal from raw materials occurs in the stage from 140 °C to 170 °C and the stage from 170 °C to 170 °C for 15 min after heating. Under the same cooking conditions, eucalyptus and acacia show different cooking efficiencies.

Due to the complexity of lignin, cellulose, and hemicellulose in the cooking reaction process [34], it is difficult to specifically go through the whole cooking stage to study the variation pattern. For the whole cooking stage, since the largest percentage of lignin removal occurs in the bulk delignification stage, it was chosen to start from this stage and study the reaction processes of different raw materials in this stage. In this stage, the reaction performances of two types of eucalyptus wood (holding times: 0, 15, 30, and 60 min) and two types of acacia wood (holding times: 0, 15, 30, 60, and 75 min) with the cooking liquor at 170 °C were tested, and the test and characterization results are shown in Table 5.

There was a good correlation between the cooking holding time and the lignin removal rate during the kinetic process. The rate constants of delignification in this stage were calculated for each raw material using an exponential function model, and as can be seen in Table 5, each of the studied samples exhibited a pseudo primary reaction, in agreement with previous similar studies [26,35]. The delignification rate constants for the four wood materials were AE, CE, VA, and IA in descending order, with the high-rate constant representing a high rate of delignification in this stage. It was divided into two different zones: the first zone represented the species that were easy to delignify (two species of eucalyptus) and the second zone represented the species that were difficult to delignify (two species of acacia). It is obvious that AE and CE have a clear advantage over VA and IA; so, the lignin of eucalyptus is a more active lignin and is easier to be delignified in this stage.

### 2.3. Effect of Lignin Side Chain Conformation on Delignification Properties of Hardwoods

#### 2.3.1. Relationship between E/T Ratio and Cooking Performance of Different Samples

**Figure 6:**
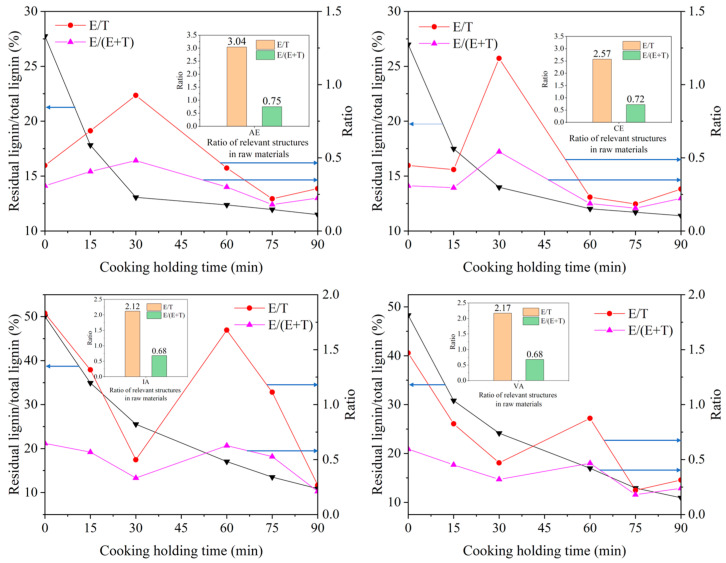
The trend of residual lignin content, E/T ratio, and E/(E + T) ratio.

The changes in the lignin side chain isomers and types of structural units of lignin of IA, VA, CE, and AE with cooking time were studied via ozonation and NBO, respectively. The trends of residual lignin content and E/T ratio are shown in Figure 6. It can be seen that the changes in E/T ratio of the four raw materials follow similar rules.

In the stage of massive delignification, the E/T ratio decreases continuously with the cooking process, and this decreasing trend continues until the lignin removal rate begins to decrease. There are two main reasons for the decrease in the E/T ratio: first, the initial E/T ratio of lignin in raw materials is high, and most of the β-*O*-4′ structure of lignin in this stage is mainly E-type (which is mainly related to the formation of initial lignin). Second, the E-type β-*O*-4′ structure of lignin is more prone to chemical reactions and thus fracture than the T-type, which is mainly related to the attack mode of nucleophilic groups in the cooking liquid. For IA and VA, the ratio of E/T keeps decreasing until 30 min of the cooking holding period. However, for CE and AE, this time point is advanced to about 15 min. Especially for AE, this time may be earlier. When this stage is over, the residual lignin content in the raw material is about 5–8%. At the same time, the E/T value of residual lignin from different raw materials is about 0.5. In other words, when a large amount of delignification occurs in the early stage of cooking, the E/T value decreases rapidly due to the proportion of E-type β-*O*-4′ spatial configuration in the raw materials and reaction characteristics. When the content of the T-type β-*O*-4′ structure is higher than that of E-type, especially when the E/T ratio is reduced to 0.5, the delignification rate starts to decrease. Instead, the E/T ratio starts to increase.

This is because a large number of E-type β-*O*-4′ structures in the delignification stages have basically finished the reaction, and the β-*O*-4′ structure of lignin removed in this stage is mainly T-type. For IA and VA, this process occurs when the holding time is 30–60 min. For CE and AE, this process occurs when the holding time is 15–30 min, and it is even earlier for AE, separately. When most of the T-type β-*O*-4′ structure lignin is removed, it means that the lignin in the raw material has basically completed the overall removal of most of the lignin; at this time, the lignin content is below 5%. When the lignin with a T-type β-*O*-4′ structure is consumed continuously, the E/T ratio also increases, indicating that the E-type β-*O*-4′ structure would replace the lignin with a T-type β-*O*-4′ structure to participate in the main cooking reaction. Therefore, the E/T ratio begins to decrease gradually in the late cooking period. When the lignin reaction is basically completed in the cooking reaction stage, the E/T ratio is about 0.3, and the lignin content of the reaction products is about 3%. For IA and VA, this process occurs when the holding time is 60–90 min. For CE and AE, this process occurs when the holding time is 30–90 min. It can be preliminarily concluded that when the value of the E/T ratio is high, especially higher than 1.0, the lignin removed via the cooking reaction is mainly of an E-type β-*O*-4′ structure. In the stage of massive delignification, when the E/T value decreased to 0.5, the T-type β-*O*-4′ structure would become the main type involved in the reaction due to the proportion of E-type and T-type. Combined with the previous results of lignin content determination, the delignification efficiency of both of the eucalyptus woods was significantly better than that of the acacia woods.

#### 2.3.2. Relationship between S/G Ratio and Cooking Performance of Different Samples

**Figure 7:**
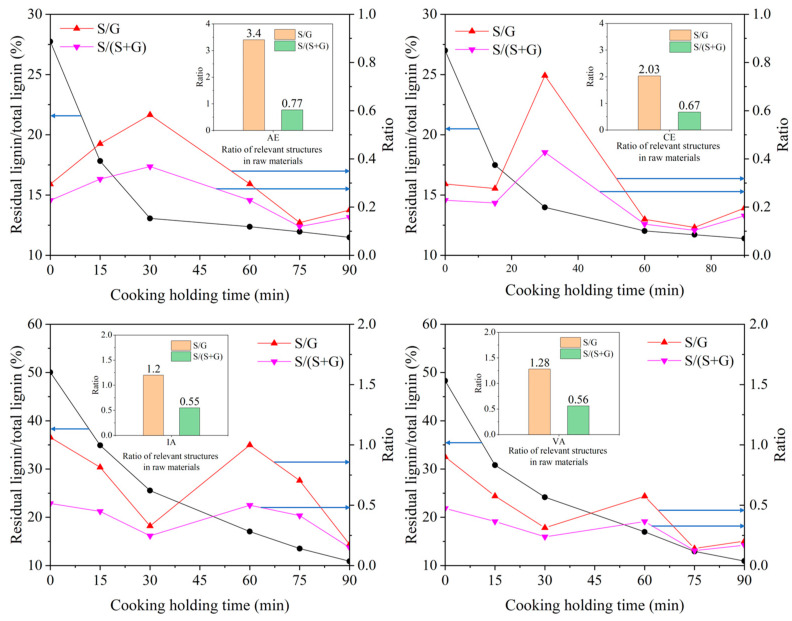
Trends of residual lignin content, S/G value, and S/(S + G) ratio.

In the β-*O*-4′ substructure of lignin, the content of the syringyl nuclei is significantly, linearly, and positively correlated with the erythro side chain structures [9,11]. Therefore, it is clear from Figure 7 that the S/G ratio has the same pattern of variation as the E/T ratio.

The presence of syringyl nuclei significantly affects the delignification rate under alkaline conditions, and the higher the content of syringyl nuclei and the E-type side chain structure in the β-*O*-4′ substructure of lignin, the easier the delignification under alkaline conditions [9]. The results of this experiment showed that during the stage of massive delignification, the S/G ratio continuously decreased with the reduction in lignin content, and this decreasing trend was maintained until the efficiency of lignin removal started to decrease. In this process, the change in the S/G ratio was similar to the changing pattern of the E/T ratio, described earlier. There were two main reasons for the reduction in the S/G ratio: firstly, the initial S/G ratio of lignin in the raw material was high, and the monomeric structure of lignin in this stage was mostly S-type, which was mainly related to the formation of the initial lignin. Secondly, under alkaline conditions, the presence of S-type nuclei obviously affected the rate of delignification, and the higher the content of S-type nuclei, the faster the rate of delignification. In this reaction stage, the S-type nuclei are the main structural units involved in the reaction, which is mainly related to the way the lignin structural monomers participate in the chemical reaction, and this change is consistent with the previous findings.

## 3. Materials and Methods

### 3.1. Materials

In October 2020, four types of hardwood chips were purchased from different places of origin, which were *Acacia crassicarpa* from Indonesia (IA), *Acacia crassicarpa* from Vietnam (VA), *Eucalyptus grandis* Hill from Yunnan Province, China (CE), and *Eucalyptus globulus* from Australia (AE). First, the raw materials were placed in a cool place to air dry, and then the screen was used to screen out the appropriate size of wood chips. The wood chips were cut into wood strips with scissors, and then pulverized via a shredder. Finally, the wood meal was passed through a 40–80 mesh iron screen, and the 40–80 mesh wood powder was obtained after the screening. After mixing evenly, the wood powder was stored in a plastic seal pocket for balance. A 3–5 g sample was extracted with ethanol/benzene (1/2) mixture solution (extraction time was 6–8 h). The extracted residue was dried at 105 °C, and the weight reduction was due to the extract. At the same time, the dryness of the raw material powder after balance was measured, and the proportion of the extract was calculated so that the extract-free sample was obtained. The extracted sample was placed in a fume hood and allowed to air dry naturally, followed by vacuum drying.

### 3.2. Methods

#### 3.2.1. Analysis of Lignin and Saccharides of Raw Materials

The contents of lignin and saccharides in the raw material were calculated referring to the method described by Borchardt [36] et al. Around 300 mg of sample was placed directly into a pressure-resistant vial to facilitate the subsequent 120 °C hydrolysis reaction in an autoclave. At the same time, 72% (*w*/*w*) sulfuric acid solution was added to it. It was stirred gently for one minute with a glass stirring bar. This step ensured that the sample was well moistened and melted, and then it was placed in a water bath at 30 °C for 1 h. It was stirred every 5–10 min during this time to ensure uniform acid distribution. Distilled water was added to the bottle at the end of the reaction, and then it was capped and allowed to mix well. At this point, the sulfuric acid concentration was 4% (*w*/*w*), and then the reaction was carried out in an autoclave at 120 °C for 60 min. The products were analyzed via high-performance liquid chromatography (Waters 1525 Analysis, Milford, MA, USA) using an Aminex HPX-87H column, 300 × 7.8 mm, with a differential refractive index detector and a mobile phase of 5 mmol/L H_2_SO_4_ solution at a flow rate of 0.6 mL/min. The column temperature was 55 °C.

#### 3.2.2. Preparation of a Series of Lignocellulosic Biomass Using Different Cooking Conditions

The traditional kraft pulping (KP) [37,38] method was used to obtain pulp in different stages of cooking. Lignin, cellulose, and hemicellulose aggregates containing different lignin contents were obtained via this program. Laboratory cooking experiments were carried out in a 2 L cooking pot. The wood strips were placed in the pot, and it was the same for four raw materials. NaOH, Na_2_S, and deionized water were mixed and added to the pot and then cooked according to the cooking schedule. The conditions used for cooking were 21% effective alkali, 25% sulfidity, and a 5:1 liquid ratio. Under the same cooking conditions as above, different cooking times were used to obtain lignin in different cooking stages. In total, the cooking periods were divided into six stages: heating up, and holding for 15, 30, 60, 75, and 90 min.

In the heating-up phase, the digester was first started and allowed to rise from room temperature to 80 °C before stopping the heating, and then the cooking pot was placed in the digester for operation. The cooking pot was driven by the digester and rotated counter-clockwise in the oil bath at 80 °C for 10 min, which made the cooking liquid and the raw wood chips be evenly mixed. Next, the heating-up procedure was started, and the time taken to control the temperature of the cooking pot from 80 to 170 °C was 90 min. The cooking samples at this point were removed to obtain the pulp for the heating-up phase of each raw material. The next operation was the same as above, and the cooking samples at 15, 30, 60, 75, and 90 min of holding time were removed in turn to obtain the pulp for each raw material with the corresponding holding period. All cooking samples were washed with water until they were neutral, dried via centrifugation, and placed in a sealed bag.

#### 3.2.3. Determination of Lignin Content

Briefly, 8 g lithium chloride (LiCl) was dissolved in 92 g dimethyl sulfoxide (DMSO) dried via molecular sieve to obtain an 8% LiCl/DMSO solvent system [39]. The samples were pre-treated via ball milling in order to improve the solubility of the samples in a LiCl/DMSO solvent system. All wood powder samples were vacuum-dried for 12 h and then the samples were ball-milled using a planetary micro-mill (Mill No. 7, Fridge, Idar-Oberstein, Germany) for 6 h. The pulp obtained via cooking required ball milling for 2 h. The ball-milled sample and LiCl/DMSO solution were mixed in a glass bottle. Then, in order to obtain a completely dissolved solution, the glass bottle was sealed, and the suspension was magnetically stirred for 24 h at room temperature. For the pulp made of four raw materials, a completely dissolved solution was obtained by further increasing the temperature to 60 °C and stirring for 2 h.

Lignocellulosic biomass LiCl/DMSO mixture was diluted 5–25 times in a volumetric flask with 8% LiCl/DMSO solution to control absorbance values in the range of 0.2 to 0.8. The 8% LiCl/DMSO solution was taken as a blank control, and the mixed LiCl/DMSO solution was put into a quartz test tube with a thickness of 1 cm and scanned in the wavelength range of 200–800 nm [40] to determine the absorbance values of the samples at characteristic absorption wavelengths [41]. The scan interval was 1 nm, the scan speed was medium, and the slit width was 1 nm. The measurement method was Abs.

#### 3.2.4. Preparation of Milled Wood Lignin Samples (MWLs) from Ground Wood

Briefly, 50 g of ball-milled wood powder was taken, and 96% dioxane (dioxane: wood meal = 10:1) was added and extracted for 48 h by stirring under light and magnetic force [40]. After stirring, the supernatant was separated via centrifugation, and the extraction residue was added to the 96% dioxane solution, then extracted via stirring for 48 h, and centrifuged. The clear liquid of two centrifugations was concentrated to dry powder via spinning at about 36 °C under reduced pressure, and the distillation flask was weighed before spinning. The residue was air-dried twice for the extraction of milled wood quality. After vacuum drying, the distilled substrate was weighed and the weight of the empty bottle was subtracted to obtain the quality difference.

A suitable amount of 90% acetic acid was added to dissolve the lignin [42]. The supernatant after acetic acid extraction was slowly added to deionized water. The solid residue after precipitation was washed 2 to 3 times with deionized water until it was neutral, and then freeze-dried and vacuum-dried. The freeze-dried and vacuum-dried product was added to an appropriate amount of 1,2-dichloroethane: ethanol (2:1, *v*/*v*) mixture until it was dissolved. After centrifugation, the supernatant was added to a large amount of 1,2-dichloroethane: ethanol (2:1, *v*/*v*) solution. The obtained precipitate was washed via centrifugation with ether 3 times and then dried in a vacuum drying oven. The purified MWLs were then obtained.

#### 3.2.5. Ozone Oxidation

**Figure 8:**
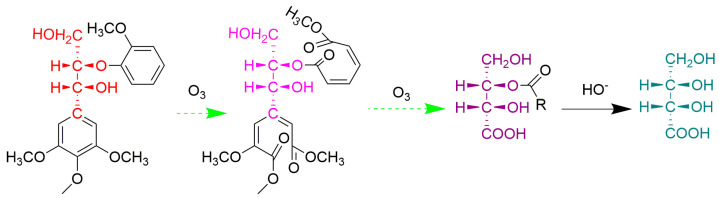
The principle of the ozone reaction of lignin dimer.

Ozonation was carried out according to the procedure reported by Akiyama et al. [4] and optimization was carried out on top of that foundation. Ozone oxidation worked mainly to destroy the benzene ring structure, which led to the opening of the benzene ring and the formation of small molecule compounds. The specific principle is shown in Figure 8. More importantly, it did not destroy the spatial structure of the original molecule.

The details of ozone oxidation are as follows: A certain amount of sample was suspended in a wide-mouth bottle with 50 mL distilled water, and a certain amount of NaBH_4_ was added. When no bubbles were produced, NaBH_4_ continued to be added, and the reaction was carried out for 30 h at room temperature with constant stirring. When the reaction time was reached, acetic acid was added to decompose the excess NaBH_4_ until no bubbles were produced. Afterward, it was filtered, washed with deionized water, and the filtered sample was collected. A sample equivalent to 20 mg lignin was weighed and suspended in a 250 mL wash cylinder containing 50 mL acetic acid: water: methanol mixture (16:3:1, *v*/*v*/*v*). Then, the ozone generator was turned on, the current was adjusted to 1.5 A, and oxygen flowed at 0.1 m^3^/h as required. After the ozone generator was stable for 30 min, the mixed gas of oxygen and ozone was passed into the washing gas cylinder. The washing gas cylinder should be immersed in an ice water bath (0 °C), and the reaction should be carried out under the condition of constant stirring. The time of ozonolysis was 120 min. After reaching the reaction time, nitrogen was immediately injected to remove the excess ozone. After the ozonolysis time had been reached, nitrogen was injected into the mixture for 1–2 min to discharge the excess ozone, and then the sodium thiosulfate solution was added to the mixture immediately, and placed there for 1 h. After being filtered, the filtrate was dried using a rotary evaporator. Distilled water was added to the dried material and dried. This procedure should be repeated twice. Erythritol: methanol (1:1, *v*/*v*) solution was added to the dried material as an internal standard. NaOH solution was added to the dried mixture, shaken well, and then nitrogen was passed into the solution for 1–2 min to eliminate oxygen. Then, the bottle was plugged tightly and left overnight at room temperature under dark conditions. Finally, the produced sample was ready to be derivatized and analyzed via gas chromatography mass spectrometry (GC-MS).

In this study, the subsequent treatment steps of ozonation were also optimized. First, the pre-reduction step was added before ozonolysis, and sodium borohydride was used to reduce the carbonyl and aldehyde groups in raw materials to hydroxyl groups, which was more conducive to the subsequent reaction. The other method was to dissolve the ozonation product obtained after rotary evaporation in pyridine, and add BSTFA at 60 °C for derivatization and determination, in order to facilitate the quantification and separation of the target product [43]. GC-MS analysis of the samples was recorded using an ISQ gas chromatograph mass spectrometer (Frontier, USA, and Japan) equipped with a thermal cracking device. A DB-5MS capillary column (30 m × 0.25 mm × 0.25 μm) was used. The injection port temperature was 250 °C. High-purity helium (99.999%) was used as the carrier gas and the maximum temperature that the column could withstand was 350 °C. The method types used for all of the sample tests were common. The injection volume for each sample was 0.4 μL, and the temperature of the vapor chamber was 250 °C. The shunt ratio was 30.0 and the non-shunt time was 1 min. The initial temperature of the column was 50 °C and the retention time at this point was 0 min. The subsequent ramp-up procedure increased the temperature from 50 °C to 250 °C at a rate of 15 °C/min and the retention time at this temperature was 15 min.

#### 3.2.6. Nitrobenzene Oxidation

In lignocellulosic materials, alkaline nitrobenzene oxidation (NBO) is one of the most important and commonly used degradation methods to characterize the lignin structure. In NBO, the lignin is degraded to aldehydes, *p*-hydroxyphenyl (H), guaiacyl (G), and syringyl (S), corresponding to *p*-hydroxybenzaldehyde (B), vanillin (V), and syringaldehyde (Sr), respectively. Their properties on the benzene ring do not change with the reaction. These structures affect the utilization of biomass in paper and bioenergy production. Alkaline nitrobenzene oxidation was carried out according to the procedure reported by Chen [44]. The reaction products were trimethylsilylated with *N* and *O*-bis (trimethylsilyl) acetamide. The products were analyzed using an ISQ gas chromatograph mass spectrometer (Frontier, USA, and Japan) equipped with a thermal cracking device. A DB-5MS capillary column (30 m × 0.25 mm × 0.25 μm) was used. Both injector and detector temperatures were 280 °C. The column temperature was kept at 150 °C for 10 min, and then programmed to rise to 250 °C at 5 °C/min. 3-Ethoxy-4-hydroxybenzaldehyde (ethylvanillin) was used as an internal standard.

#### 3.2.7. FTIR Spectra

FTIR spectra of the four materials were recorded using a VERTEX 80 V FTIR spectrometer (Bruker, Germany). The MWLs were mixed with KBr. The scan area was 4000−400 cm^−1^. The scan resolution was 4 cm^−1^.

#### 3.2.8. 2D HSQC NMR

The MWLs (50 mg) were dissolved in 0.5 mL of deuterated dimethyl sulfoxide (DMSO-d6) according to the method previously described [45]. The central solvent peak was used as the internal reference (δ_C_/δ_H_ 39.5/2.50) [46]. The 2D NMR spectra of the raw material were recorded at 25 °C using an AVANCE III 600 MHz instrument (Bruker, Switzerland), which contains 3 RF transmit channels and a receiving channel capable of detection in normal and reverse mode. It is equipped with a deuteron-locked field and deuteron-gradient automatic homogenization accessory, a z-directional pulsed-gradient field, and a cryogenically cooled 5 mm TCI z-gradient triple resonance probe. The experiments used Bruker’s “hsqcetgpsp.2” adiabatic pulse program with spectral widths from 0 to 16 ppm (9615 Hz) and from 0 to 165 ppm (24,900 Hz) for the ^1^H- and ^13^C-dimensions. The number of collected complex points was 2048 for the ^1^H-dimension with a recycle delay of 1.5 s. The number of transients was 64, and 256 time increments were recorded in the ^13^C-dimension. The ^1^*J*CH used was 145 Hz. Processing used typical matched Gaussian apodization in the ^1^H-dimension and squared cosine-bell apodization in the ^13^C-dimension. Prior to Fourier transformation, the data matrices were zero-filled to 1024 points in the ^13^C-dimension.

## 4. Conclusions

The structures of the four raw materials (AE, CE, IA, and VA), which are so similar in their component composition, diverged into two groups, as seen via the results of the study. The results obtained via UV spectroscopy show that the delignification efficiency of AE and CE is significantly higher than that of IA and VA, and most of the lignin is basically removed before the node of cooking and holding time of 15 min, while that of IA and VA is around the node of 30 min. The results of 2D HSQC NMR, ozonation, and NBO show that the lignin of AE and CE contains more of a β-*O*-4′ structure, mainly the E-type configuration, and the E/T and S/G ratios are relatively higher. The E/T ratios of the four raw materials all go through three stages, first decreasing, then increasing, and finally decreasing, and the first minimum value was at the turning point of their respective lignin removal rates. Meanwhile, the S/G ratio has almost the same variation pattern as this. The E/T and S/G ratios of lignin will affect the efficiency of the cooking reaction. During the cooking process, the changes in the E/T and S/G ratios are closely related to the change in lignin content and removal rate, which lead to different cooking efficiencies of different raw materials. Therefore, the pulping efficiency of different raw materials can be improved via different technological means.

## Figures and Tables

**Table 1:** Chemical composition of raw materials.

Sample	Lignin			Carbohydrate			NBO	Ozonation
	KL	ASL	TL	Glucan	Xylan	Arabinan	S/V/H	E/T
AE	18.04 ± 0.90	6.81 ± 0.61	24.85 ± 0.7	41.37 ± 0.51	17.48 ± 0.14	0.64 ± 0.04	75/24/1	2.54
CE	22.08 ± 0.21	1.74 ± 0.03	23.82 ± 0.24	43.7 ± 0.47	13.66 ± 0.25	0.59 ± 0.06	67/32/0	2.63
IA	22.54 ± 0.08	1.67 ± 0.04	24.21 ± 0.12	49.37 ± 0.23	13.69 ± 0.46	-	54/45/1	2.13
VA	20.68 ± 0.02	3.43 ± 0.07	24.11 ± 0.1	44.14 ± 0.11	15.89 ± 0.17	-	52/47/1	2.17

**Table 2:** Band assignments for the FT-IR spectra of MWLs.

Wavelength (cm^−1^)	Assignment
3430	Stretching vibration of –OH (phenolic and alcoholic hydroxyl)
2937	C–H stretching vibration in the aromatic methoxyl
2842	C–H stretching in methyl and methylene of the side chains
1727	Stretching vibration of non-conjugate C=O
1653	Stretching vibration of conjugate C=O
1594	Stretching vibration of benzene ring
1506	Stretching vibration of benzene ring
1462	Bending vibration of C–H (CH_2_, CH_3_)
1423	Stretching vibration of benzene ring
1329	Stretching vibration of C–O in S-unit
1267	Stretching vibration of C–O in G-unit
1225	Stretching vibration of C–O in S-unit
1124	In-plane bending vibration of C–H in benzene ring of S-unit
1031	Stretching vibration of C–O (alcoholic hydroxyl and alkyl ether)

**Table 3:** Assignment of ^1^H-^13^C correlation peaks in the 2D NMR spectra of MWLs.

δ_H_ (ppm)	δ_C_ (ppm)	Label	Assignment
2.78	59.78	C_β_	C_β_-H_β_ in spirodienones (C)
3.06	53.48	B_β_	C_β_-H_β_ in β-β′ (resinol) substructures (B)
3.25	59.78	A_γ_	C_γ_-H_γ_ in β-*O*-4′ substructure (A)
3.50	75.54	X_4_	C_4_-H_4_ in β-*D*-xylopyranoside (X)
3.63	59.78	A_γ_	C_γ_-H_γ_ in β-*O*-4′ substructure (A)
3.75	55.73	OMe	C-H in methoxyls (OMe)
3.85	71.04	B_γ_	C_γ_-H_γ_ in β-β′ (resinol) substructures (B)
4.13	85.90	A_β_(S)	C_β_-H_β_ in β-*O*-4′ substructures (A) linked to S(Erythro)
4.19	71.04	B_γ_	C_γ_-H_γ_ in β-β′ (resinol) substructures (B)
4.29	84.10	A_β_(G)	C_β_-H_β_ in β-*O*-4′ unit (A) linked to a G-unit
4.32	83.65	B_α_	C_α_-H_α_ in β-β′ (resinol) substructures (B)
4.48	72.84	A_α_	C_α_-H_α_ in β-*O*-4′ unit (A)
4.69	85.00	B_α_	C_α_-H_α_ in β-β′ resinol substructures (B)
4.79	71.04	A_α_(G)	C_α_-H_α_ in β-*O*-4′ unit (A) linked to a G-unit (A)
4.88	71.94	A_α_(S)	C_α_-H_α_ in β-*O*-4′ unit (A) linked to an S-unit (A)
5.10	81.40	C_α_	C_α_-H_α_ in spirodienones (C)
5.48/5.64	86.80	D_α_	C_α_-H_α_ in phenylcoumaran substructure (D)
6.21	113.37	*p*CA_8_	C_8_-H_8_ in *p*-coumarate (*p*CA)
6.25	128.68	F_ɑ_	C_α_-H_α_ in *p*-hydroxycinnamyl alcohol (F)
6.48	128.68	F_β_	C_β_-H_β_ in *p*-hydroxycinnamyl alcohol (F)
6.72	103.91	S_2,6_	C_2,6_-H_2,6_ in syringyl units (S)
6.73	114.72	G_5_	C_5_-H_5_ in guaiacyl units (G)
6.79	126.43	J_β_	C_β_-H_β_ in cinnamyl aldehyde end-groups (J)
6.80/6.85	118.77	G_6_	C_6_-H_6_ in guaiacyl units (G)
6.98	115.17	A_α_	C_5_-H_5_ in etherified guaiacyl units (G)
7.01/7.34	111.12/112.47	G_2_	C_2_-H_2_ in guaiacyl units (G)
7.20	127.78	H_2,6_	C_2,6_-H_2,6_ in H units (H)
7.22	106.61	S′_2,6_	C_2,6_-H_2,6_, C(α)=O in syringyl units (S′)
7.23	123.27	FA_6_	C_6_-H_6_ in ferulate (FA)
7.33	118.77	G_6_	C_6_-H_6_ in guaiacyl units (G)
7.65	153.89	J_α_	C_α_-H_α_ in cinnamyl aldehyde end-groups (J)

**Table 4:** Semi-quantitative calculation of ^1^H-^13^C HSQC NMR (based on C_α_).

Lignin Aromatic Units (%)	AE	CE	IA	VA
G	23.85%	33.01%	44.79%	40.91%
S	74.95%	66.99%	53.53%	52.18%
H	1.20%	0.00%	1.67%	6.91%
S/G ratio	3.40	2.03	1.20	1.28
Lignin interunit linkages (%)				
β-*O*-4′ (A)	69.72%	65.61%	61.96%	60.20%
Resinol (B)	26.19%	13.86%	22.18%	27.17%
Spirodienones (C)	4.61%	6.30%	3.25%	3.95%
Phenylcoumaran (D)	3.51%	7.42%	8.48%	8.68%
Dibenzodioxocin substructutres (E)	-	2.82%	-	-
*p*-hydroxycinamates (%)				
*p*-coumarates (PCA) and *p*-hydroxycinnamyl alcohol (F)	3.93%	3.58%	4.32%	5.81%
Ferulate (FA)	1.04%	3.43%	1.91%	1.82%
*p*-coumarates and *p*-ydroxycinnamyl alcohol/ferulate ratio	3.79	1.04	2.26	3.19
Lignin end-groups				
Cinnamyl aldehyde end-groups (J)	3.31%	3.31%	6.97%	7.71%

**Table 5:** Regression equations and rate constants during bulk delignification stage.

Samples	Regression Equation	Rate Constant (100 × 1/min)	R^2^
AE	y = 17.61exp(−x/14.29) + 12.97	7.01	0.99
CE	y = 18.17exp(−x/15.49) + 13.97	6.46	0.99
VA	y = 39.80exp(−x/25.40) + 13.83	3.94	0.99
IA	y = 48.55exp(−x/31.63) + 12.70	3.61	0.99

## Data Availability

Not applicable.

## Sample Availability

Not available.
